# Parvalbumin interneurons: the dark and bright sides of a key playmaker of neural circuits and behavior

**DOI:** 10.3389/fncel.2025.1738489

**Published:** 2025-12-19

**Authors:** Eesha Wirk, Charles Quairiaux, Thomas Marissal

**Affiliations:** 1 INMED, INSERM, Aix-Marseille University, Marseille, France; 2 Department of Basic Neuroscience, Faculty of Medicine, University of Geneva, Geneva, Switzerland

**Keywords:** behavior, circuits, cortex, disease, health, hippocampus, memory, parvalbumin interneurons

## Abstract

With their morphological and electrophysiological properties as well as exceptional connectivity, parvalbumin interneurons play a major role in the dynamics of the neural circuits of the hippocampus and cortex, along with associated cognitive functions. Their dysfunction, which is sometimes reversible, contributes to significant disruptions in network activity and behavioral deficits related to various diseases such as epilepsies or neuropsychiatric disorders. In this Mini Review, we present these parvalbumin interneurons, their characteristics, pathophysiological roles, and propose avenues for future investigations.

## 1 Introduction

Parvalbumin interneurons (PVIs) constitute a small fraction of the total neurons of the hippocampus and cortex (2.5%–10%) (Houser, 2007; Bezaire and Soltesz, 2013; Druga et al., 2023). Even within the heterogeneous population of GABAergic inhibitory cells, PVIs are present in numbers comparable to or even lower than other subtypes, such as interneurons expressing VIP or NPY in the hippocampus (Rudy et al., 2011; Pelkey et al., 2017). However, PVIs receive disproportionate attention, which is explained by their many remarkable features in healthy conditions, that enable them to orchestrate network dynamics and control associated behaviors. Consequently, the artificial manipulation (using cell-specific genetic tools) or the alterations (in rodent models related to diseases) of PVI properties can lead to disruption of neuronal activity, the onset of epileptic seizures, and failure of cognitive functions. Here, we describe the exceptional characteristics of PVIs, their contribution to normal function and disease, as well as therapeutic approaches that target them.

## 2 Hippocampal and cortical PVIs: from the physiology to disease

### 2.1 PVIs under healthy condition

**FIGURE 1:**
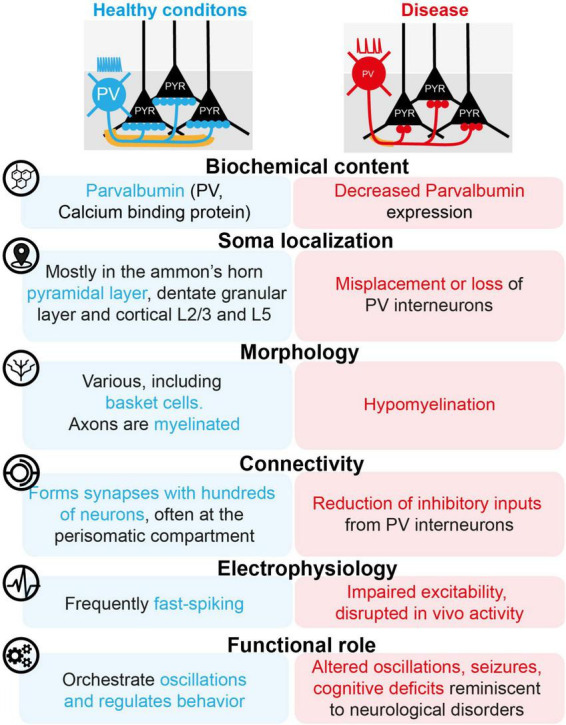
The dark and bright sides of PVIs.

With their somata particularly concentrated in certain subregions, such as the stratum pyramidale of the Ammon horns, the edges of the granular layer in the dentate gyrus, and layers L2/3 and L5 of the cortex, PVIs display many distinctive properties, detailed below according to the traditional nomenclature used to classify interneurons (Ascoli et al., 2008; Figure 1).

#### 2.1.1 PVIs morphologies

Parvalbumin interneurons in the hippocampus and cortex are divided into several subcategories, based on their axonal arborization. The two best-documented categories are basket cells (or BCs) and chandelier cells (also called “axo-axonic” cells, or AACs).

In Ammon’s Horn of the hippocampus, parvalbumin-expressing BCs have a large pyramid or spindle-shaped soma. Generally lacking spines, their dendrites extend from the alveus to the stratum lacunosum-moleculare. Emerging from the soma or a primary dendrite, their axons form numerous collaterals in the pyramidal layer, giving it a basket-like appearance, with some incursions into the strata oriens and radiatum (Pelkey et al., 2017).

In the dentate gyrus of the hippocampus, BCs have a similar morphology, extending their axons into the granular layer and their dendrites from the outer molecular layer to the hilus (Koh et al., 1995). Unlike Ammon’s Horn, dentate BCs are covered with dendritic spines, the density of which is regulated by experience (Kaufhold et al., 2024).

In the cortex, parvalbumin-positive BCs have a generally multipolar dendrite and an axon that forms a plexus, which can be large and cover several cortical layers and columns or be smaller and restricted to a single layer (Kubota, 2014; Tremblay et al., 2016).

In CA1, AACs have axons that branch out into the pyramidal layer and the superficial part of the stratum oriens (Li et al., 1992). The main branches often extend horizontally, with vertically arranged terminals and rows of synaptic boutons, giving them a distinctive candelabrum appearance (Pelkey et al., 2017). Their dendrites extend either radially from the alveus to the stratum lacunosum-moleculare (Li et al., 1992) or horizontally, spreading exclusively in the stratum oriens, parallel to the pyramidal layer, over several hundred micrometers (Ganter et al., 2004). AACs with similar morphology have been identified in CA3, the dentate gyrus, and the cortex (Somogyi, 1977; Buhl et al., 1994; Viney et al., 2013).

Parvalbumin can be expressed by other morphological subtypes, such as the bistratified cells (BiS) of Ammon’s horns (Halasy et al., 1996; Pelkey et al., 2017), the O-LM cells (Oriens lacunosum-moleculare) of Ammon’s horns and their equivalent in the dentate gyrus, called “HIPP” for “hilar perforant path associated cells” (McBain et al., 1994; Freund and Buzsáki, 1996; Pelkey et al., 2017), the multipolar bursting cells of the cortex (Blatow et al., 2003), as well as long-distance projection neurons of the hippocampus (Jinno et al., 2007; Wick et al., 2017; Yen et al., 2022) and the cortex (Bertero et al., 2020). It should be noted that the axons of PVIs in the hippocampus are often surrounded by a myelin sheath (Stedehouder et al., 2017).

#### 2.1.2 Connectivity

While some PVI subtypes preferentially form synapses on the dendrites of excitatory cells, such as hippocampal O-LM or BiSs (Pelkey et al., 2017), the majority of hippocampal and cortical PVIs target the perisomatic compartment of numerous pyramidal cells and efficiently control their output (Freund and Katona, 2007; Stokes and Isaacson, 2010). AACs exclusively contact the initial segment of the axon of hundreds of pyramids in the hippocampus (Li et al., 1992) and cortex (Wang Y. et al., 2016). Parvalbumin-expressing BCs form synapses with the soma and proximal dendrites of 1500–2500 pyramids in CA1 of the hippocampus, forming half a dozen synapses with each of them (Freund and Buzsáki, 1996; Pelkey et al., 2017). In the cortex, each BC innervates 200–1000 pyramids with 5–15 terminal boutons (Karube et al., 2004; Kubota, 2014). The extensive dendrites of CA1 BCs receive a large number of convergent excitatory inputs, both local (from pyramidal neurons) and distant (e.g., entorhinal cortex) (Gulyás et al., 1999; Tukker et al., 2013). Interestingly, parvalbumin-containing BCs inhibit the deep pyramids of CA1 more strongly but receive more excitation from pyramids located in the superficial part of the pyramidal stratum. Similarly, PVIs tend to innervate pyramidal neurons projecting to the amygdala but receive preferential excitation from pyramids projecting to the prefrontal cortex (Lee et al., 2014). In the cortex and other structures such as the presubiculum (Peng et al., 2021), although observations suggest that PVIs randomly innervate surrounding pyramidal cells and receive excitatory inputs from most nearby pyramids, it appears that the strongest reciprocal connections occur between PVIs and pyramids participating in the same functional process (Packer and Yuste, 2011; Znamenskiy et al., 2024).

#### 2.1.3 Biochemical markers

In PVIs, the expression levels of the calcium-binding parvalbumin protein itself differ depending on the morphological subtype. Thus, parvalbumin labeling is weaker in BiSs and O-LM cells in the hippocampus than in AACs or BCs (Ferraguti et al., 2004). Key to PVI function (Zhang et al., 2025), parvalbumin expression vary depending on experience (Donato et al., 2013) or neuronal activity (Patz et al., 2004; Rupert and Shea, 2022). This form of plasticity appears to be related to perineuronal nets (marked by aggrecan or vicia villosa agglutinin) that mainly envelop PVIs (Yamada et al., 2015).

In addition, PVIs, particularly BCs in the hippocampus and cortex, are characterized by the compartmentalized expression of a combination of proteins associated with rapid, strong, and efficient signaling (Hu et al., 2014). Thus, PVI possesses calcium-permeable AMPA-type glutamatergic receptors that lacks GluA2, but contains GluA1 and GluA4 subunits, as well as GABRA1-containing GABAergic receptors, which are associated with fast-acting excitatory and inhibitory postsynaptic currents (Geiger et al., 1995, 1997; Bartos et al., 2002; Hong et al., 2024). Similarly, the supercritical density of NaV1.1 and NaV1.6 sodium channels along axons, combined with myelination (Micheva et al., 2021), allows for rapid propagation of action potentials (Ogiwara et al., 2007; Lorincz and Nusser, 2008; Hu et al., 2014). At the end of the chain, presynaptic calcium channels of the Cav2.1 or P/Q types, closely coupled to the calcium sensor Synaptotagmin 2, enable rapid and precise secretion of the neurotransmitter GABA (Hefft and Jonas, 2005; Pang et al., 2006; Zaitsev et al., 2007; Bucurenciu et al., 2010; Eggermann et al., 2011; Sommeijer and Levelt, 2012; Rossignol et al., 2013; Lee et al., 2014).

Interestingly, certain protein markers can be used to distinguish between morphological subtypes of PVIs, particularly in the hippocampus. For example, somatostatin has been identified in BiSs and O-LMs (Klausberger et al., 2003, 2004), SATB1 in BCs and BiSs (Viney et al., 2013), NPY in BiSs (Klausberger et al., 2003), and mGluR1α in O-LMs (Ferraguti et al., 2004). AACs of the dentate gyrus are positive for PTHLH and Unc5b (Paul et al., 2017; Proddutur et al., 2023).

#### 2.1.4 Electrophysiological properties and *in vivo* activity

Parvalbumin BCs of the cortex and hippocampus, as well as BiS and AACs in the hippocampus, are often correlated with a fast-spiking pattern (Hu et al., 2014). This mode is defined as the ability of PVIs recorded *ex vivo* to generate, following a membrane depolarization plateau, a continuous train of high-frequency action potentials without accommodation (Wang B. et al., 2016). A “stutter firing” pattern, characterized by bursts of action potentials separated by random periods of silence, has been observed in some BiSs (Pawelzik et al., 2002). With distinct electrophysiological characteristics (Tricoire et al., 2011), including strong adaptation of action potential discharge, hippocampal O-LMs rarely exceed an action potential frequency of 50 Hz (Pelkey et al., 2017). The preferential discharge of this type of interneuron occurs in the theta band (Gloveli et al., 2005), in a kainate receptor-dependent manner (Goldin et al., 2007). In the cortex, the multipolar bursting cells are distinguished by an initial burst of action potentials in response to a depolarizing step (Blatow et al., 2003).

Hippocampal PVI subpopulations can be differentiated *in vivo* according to the spatiotemporal dynamics of their activity in relation to oscillations, which play a role in the computation of behavior (Klausberger and Somogyi, 2008; Fernandez-Ruiz et al., 2023; Huang et al., 2024) and are found to be altered under pathological conditions (Uhlhaas and Singer, 2010). For instance, in awake animals, CA1 AACs fire preferentially during the middle of the descending phases of running-associated theta rhythms (5–10 Hz), while parvalbumin-expressing BCs and BiSs discharge later (Varga et al., 2014). During fast oscillations such as gamma related to running periods (25–90 Hz) and ripples recorded during rest (90–200 Hz), BCs preferentially discharge earliest during oscillatory cycles, followed by BiSs, AACs and O-LM cells (Royer et al., 2012; Varga et al., 2012, 2014; Viney et al., 2013). Double-projecting cells, a fraction of which express parvalbumin, discharge during the trough of theta cycles and just after pyramidal neurons during gamma waves recorded in anesthetized animals (Jinno et al., 2007). In the CA2 and CA3 regions of the hippocampus, PVIs also participate in ripple, theta, and gamma oscillations, but with a different discharge timing than those in CA1 (Tukker et al., 2013; Viney et al., 2013).

#### 2.1.5 Functional properties

The development of genetic tools, based on Cre recombinase (Tsien et al., 1996), which allow selective targeting of biochemical subtypes of inhibitory neurons (Taniguchi et al., 2011) combined with strategies for manipulating neuronal activity using light with optogenetics (Boyden et al., 2005; Deisseroth, 2011) or under the effect of an inert ligand using chemogenetics (Armbruster et al., 2007; Roth, 2016), has enabled the investigation of the functional role of PVI (Raven and Aton, 2021; Tzilivaki et al., 2023).

In the hippocampus, the activity of PVIs controls the synchronization and timing of pyramidal cell firing, as well as the emergence of ripple, theta, or gamma oscillatory activity (Korotkova et al., 2010; Royer et al., 2012; Nguyen et al., 2014; Amilhon et al., 2015; Ognjanovski et al., 2017; Xia et al., 2017; Antonoudiou et al., 2020). Thus, PVIs in the hippocampus contribute substantially to spatial and working memory, memory consolidation (Korotkova et al., 2010; Donato et al., 2013; Xia et al., 2017), representation of novelty (Hainmueller et al., 2024), sensorimotor gating (Nguyen et al., 2014), and control of anxiety behavior (Tiwari et al., 2024; Volitaki et al., 2024). Interestingly, different facets of the same cognitive process are performed by distinct subpopulations of PVIs (Donato et al., 2015; Hainmueller et al., 2024).

Similarly, cortical PVIs promote the synchronization of excitatory cells (Jang et al., 2020), narrow the temporal windows of pyramidal neuron response to sensory afferent (Pedroncini et al., 2024), and orchestrate oscillations (Cardin et al., 2009; Sohal et al., 2009). Consequently, PVIs contributes to a wide variety of cortical functions, such as sensory processing (Yang et al., 2017), memory (Xia et al., 2017), social discrimination (Deng et al., 2019), emotion recognition (Fujima et al., 2025), avoidance behaviors (Ho et al., 2025), and attention (Kim et al., 2016).

In conclusion, PVIs display specific morphophysiological characteristics that enable them to act as essential components of the networks. However, these key interneurons are highly vulnerable to pathological factors (Ruden et al., 2021) and their dysfunction can have harmful effects on the functions of the hippocampus or cortex.

### 2.2 PVIs under pathological conditions

#### 2.2.1 PVIs under artificial manipulation conditions

It is possible to use genetic tools to manipulate the molecular and electrophysiological properties or connectivity of PVIs in the hippocampus and cortex of mice in order to render them dysfunctional. These disruptions are sufficient to cause activity and network disorders like those observed in neurological disorders such as schizophrenia, autism, and epilepsy.

Thus, chemogenetic or optogenetic inhibition (Nguyen et al., 2014; Hu et al., 2025), depletion of parvalbumin expression (Wöhr et al., 2015), mitochondrial dysfunction (Inan et al., 2016), or deletions of Erbb4 (del Pino et al., 2013), D2-type dopamine receptors (Tomasella et al., 2018), type 5 metabotropic glutamate receptors (Barnes et al., 2015) or NMDA-type glutamate receptors (Korotkova et al., 2010) specifically in PVIs in the hippocampus or cortex lead to disturbances in oscillatory dynamics (e.g., increased or decreased theta and gamma activity) and to cognitive deficits (e.g., memory deficits, impaired locomotion, abnormal emotional and social behavior, impaired sensory-motor gating) that mimic symptoms identified in patients with schizophrenia or autism. Even more spectacularly, permanent silencing of PVIs in the subiculum, a region of the hippocampal formation, is sufficient to induce recurrent spontaneous limbic seizures in mice, a pathological feature reminiscent of temporal lobe epilepsy (Drexel et al., 2017).

Taken together, these data suggest that dysfunction of PVIs was sufficient to cause the development of symptoms associated with neurological diseases. This prompted the scientific community to take the following step: to investigate models that accurately reproduce the symptoms of neurological diseases to determine whether PVIs in the hippocampus and cortex were altered and whether they could represent a valid target for more specific therapeutic strategies.

#### 2.2.2 Parvalbumin interneuron in disease models

Parvalbumin interneurons dysfunction has been identified in the cortex and hippocampus of many models that reliably reproduce the causes of neurological diseases (environmental, genetic, or a combination of both) as well as the symptoms identified in patients.

Thus, the pathological features of neurodevelopmental disorders such as autism and schizophrenia can be mimicked in rodents, for instance by perinatal immune activation (reproducing a microbial infection during development) or by the deletion of the DISC1 gene (linked to schizophrenia) or FMR1 gene (Fragile X syndrome) or the 22q11.2 locus (DiGeorge syndrome). In addition to frequent alterations in rhythmic activity and behavior, these models are often correlated with a disruption of the properties of PVIs (Figure 1), such as a loss of PVIs (Pignataro et al., 2023), a change in the expression and plasticity of parvalbumin itself (Sauer et al., 2015; Mukherjee et al., 2019), a reduction in the expression of ion channels (Qi et al., 2025), a reduction in the number of excitatory inputs received by PVIs (Sauer et al., 2015), a reduction in the number of inhibitory inputs received by pyramids from PVIs (Sauer et al., 2015), as well as misplacement (Meechan et al., 2012), hypomyelination (Maas et al., 2020; He et al., 2025), disruption of *ex vivo* excitability (Marissal et al., 2018; Hijazi et al., 2023), and reduction of sensory-evoked activity *in vivo* (Goel et al., 2018). Interestingly, specific chemoactivation of PVIs is sufficient to restore alterations in cortical and hippocampal network activity *in vivo*, as well as cognitive alterations in mouse models of environmentally or genetically induced neuropsychiatric disorders (Goel et al., 2018; Marissal et al., 2018; Mukherjee et al., 2019; Arime et al., 2023; Pignataro et al., 2023).

In the case of epilepsy, temporal lobe epilepsy (TLE) models are probably the most commonly used. These models are often based on an insult in the form of prolonged seizures (or Status Epilepticus) induced by the administration of kainate or pilocarpine. After a latency this leads to the emergence of epileptic seizures (primarily in the in the hippocampus, which is the main epileptic focus in TLE), and behavioral comorbidities. In these models, many alterations affect the PVIs of the hippocampus. Thus, some of the PVIs degenerate during the latent phases of the disease (Dinocourt et al., 2003), although other publications suggest that they may be relatively spared (Shuman et al., 2020; Matringhen et al., 2025). The survivors undergo changes in their morphological and electrophysiological properties. This is reflected in particular by the sprouting of the axons of commissurally-projecting PVIs (Wick et al., 2017) and by the decrease in their excitability in the dentate gyrus of TLE mouse models (Proddutur et al., 2023). Interestingly, a decrease in the excitability of PVIs in the hippocampus and cortex has also been found in mouse models of genetic forms of epilepsy (e.g., deletion of voltage-gated sodium channel NaV1.1 linked to Dravet syndrome) (Tai et al., 2014; Favero et al., 2018). On this basis, several therapeutic strategies have been tested to compensate for the loss of interneurons or restore the properties of PVIs (Marissal, 2021). For example, the transplantation of stem cells from the medial ganglionic eminence (Upadhya et al., 2019), a substantial proportion of which differentiate into PVIs, reduces seizures and improves the behavior of mice. Similarly, optogenetic stimulation of PVIs, sometimes coupled with a closed-loop system (Krook-Magnuson et al., 2013), can have a beneficial effect on seizures and behavioral deficits (Kim et al., 2020), although activation of PVIs can also have paradoxically pro-epileptic effects (Lévesque et al., 2019).

## 3 Discussion

Parvalbumin interneurons possess exceptional morphophysiological properties that enable them to contribute significantly to the dynamics of cortical and hippocampal networks, as well as to behavior. Their importance in healthy conditions partly explains why their malfunction is frequently found to be associated with disease.

However, the respective pathophysiological roles of each heterogeneous subtypes of PVIs are poorly understood and should be explored in the future. Recently, tools have become available to selectively target certain subtypes, such as AACs using strategies based on the PTHLH or Unc5b markers (Raudales et al., 2024), and enabled the identification of the changes undergone by AACs after an epileptic insult (Proddutur et al., 2023).

Moreover, it remains to be determined how PVI dysfunction is affected by and affects other elements of the inhibitory microcircuits of the hippocampus and cortex in pathological conditions. Computational and experimental data suggest that PVIs dynamically cooperate under non-pathological conditions with other interneuron subtypes such as calretinin-, VIP-, somatostatin, or CCK-containing interneurons to modulate cortical and hippocampal plasticity, activity and behavior in a manner dependent on the context or behavioral state (Wang et al., 2004; Jang et al., 2020; Udakis et al., 2020; Dudok et al., 2021; Bos et al., 2025; Onorato et al., 2025; Parker et al., 2025). How this “division of labor” between interneurons is disrupted in conditions of disease is an important avenue for investigation with the aim of developing more specific and effective therapeutic strategies.

## References

[B1] Amilhon B. Huh C. Y. L. Manseau F. Ducharme G. Nichol H. Adamantidis A. (2015). Parvalbumin interneurons of hippocampus tune population activity at theta frequency. *Neuron* 86 1277–1289. 10.1016/j.neuron.2015.05.027 26050044

[B2] Antonoudiou P. Tan Y. L. Kontou G. Upton A. L. Mann E. O. (2020). Parvalbumin and somatostatin interneurons contribute to the generation of hippocampal gamma oscillations. *J. Neurosci.* 40 7668–7687. 10.1523/JNEUROSCI.0261-20.2020 32859716 PMC7531548

[B3] Arime Y. Saitoh Y. Ishikawa M. Kamiyoshihara C. Uchida Y. Fujii K. (2023). Activation of prefrontal parvalbumin interneurons ameliorates working memory deficit even under clinically comparable antipsychotic treatment in a mouse model of schizophrenia. *Neuropsychopharmacology* 49:720. 10.1038/s41386-023-01769-z 38049583 PMC10876596

[B4] Armbruster B. N. Li X. Pausch M. H. Herlitze S. Roth B. L. (2007). Evolving the lock to fit the key to create a family of G protein-coupled receptors potently activated by an inert ligand. *Proc. Natl. Acad. Sci. U. S. A.* 104 5163–5168. 10.1073/pnas.0700293104 17360345 PMC1829280

[B5] Ascoli G. A. Alonso-Nanclares L. Anderson S. A. Barrionuevo G. Benavides-Piccione R. Burkhalter A. (2008). Petilla terminology: Nomenclature of features of GABAergic interneurons of the cerebral cortex. *Nat. Rev. Neurosci.* 9 557–568. 10.1038/nrn2402 18568015 PMC2868386

[B6] Barnes S. Pinto-Duarte A. Kappe A. Zembrzycki A. Metzler A. Mukamel E. (2015). Disruption of mGluR5 in parvalbumin-positive interneurons induces core features of neurodevelopmental disorders. *Mol. Psychiatry* 20 1161–1172. 10.1038/mp.2015.113 26260494 PMC4583365

[B7] Bartos M. Vida I. Frotscher M. Meyer A. Monyer H. Geiger J. R. P. (2002). Fast synaptic inhibition promotes synchronized gamma oscillations in hippocampal interneuron networks. *Proc. Natl. Acad. Sci. U. S. A.* 99 13222–13227. 10.1073/pnas.192233099 12235359 PMC130614

[B8] Bertero A. Zurita H. Normandin M. Apicella A. J. (2020). Auditory long-range parvalbumin cortico-striatal neurons. *Front. Neural Circuits* 14:45. 10.3389/fncir.2020.00045 32792912 PMC7390902

[B9] Bezaire M. J. Soltesz I. (2013). Quantitative assessment of CA1 local circuits: Knowledge base for interneuron-pyramidal cell connectivity. *Hippocampus* 23 751–785. 10.1002/hipo.22141 23674373 PMC3775914

[B10] Blatow M. Rozov A. Katona I. Hormuzdi S. G. Meyer A. H. Whittington M. A. (2003). A novel network of multipolar bursting interneurons generates theta frequency oscillations in neocortex. *Neuron* 38 805–817. 10.1016/S0896-6273(03)00300-3 12797964

[B11] Bos H. Miehl C. Oswald A.-M. M. Doiron B. (2025). Untangling stability and gain modulation in cortical circuits with multiple interneuron classes. *eLife* 13:R99808. 10.7554/eLife.99808 40304591 PMC12043317

[B12] Boyden E. S. Zhang F. Bamberg E. Nagel G. Deisseroth K. (2005). Millisecond-timescale, genetically targeted optical control of neural activity. *Nat. Neurosci.* 8 1263–1268. 10.1038/nn1525 16116447

[B13] Bucurenciu I. Bischofberger J. Jonas P. (2010). A small number of open Ca2+ channels trigger transmitter release at a central GABAergic synapse. *Nat. Neurosci.* 13 19–21. 10.1038/nn.2461 20010820

[B14] Buhl E. H. Han Z. S. Lorinczi Z. Stezhka V. V. Karnup S. V. Somogyi P. (1994). Physiological properties of anatomically identified axo-axonic cells in the rat hippocampus. *J. Neurophysiol.* 71 1289–1307. 10.1152/jn.1994.71.4.1289 8035215

[B15] Cardin J. A. Carlén M. Meletis K. Knoblich U. Zhang F. Deisseroth K. (2009). Driving fast-spiking cells induces gamma rhythm and controls sensory responses. *Nature* 459 663–667. 10.1038/nature08002 19396156 PMC3655711

[B16] Deisseroth K. (2011). Optogenetics. *Nat. Methods* 8 26–29. 10.1038/nmeth.f.324 21191368 PMC6814250

[B17] del Pino I. García-Frigola C. Dehorter N. Brotons-Mas J. R. Alvarez-Salvado E. Martínez (2013). Erbb4 deletion from fast-spiking interneurons causes schizophrenia-like phenotypes. *Neuron* 79 1152–1168. 10.1016/j.neuron.2013.07.010 24050403

[B18] Deng X. Gu L. Sui N. Guo J. Liang J. (2019). Parvalbumin interneuron in the ventral hippocampus functions as a discriminator in social memory. *Proc. Natl. Acad. Sci. U. S. A.* 116:16583. 10.1073/pnas.1819133116 31358646 PMC6697894

[B19] Dinocourt C. Petanjek Z. Freund T. F. Ben-Ari Y. Esclapez M. (2003). Loss of interneurons innervating pyramidal cell dendrites and axon initial segments in the CA1 region of the hippocampus following pilocarpine-induced seizures. *J. Comp. Neurol.* 459 407–425. 10.1002/cne.10622 12687707

[B20] Donato F. Chowdhury A. Lahr M. Caroni P. (2015). Early- and late-born parvalbumin basket cell subpopulations exhibiting distinct regulation and roles in learning. *Neuron* 85 770–786. 10.1016/j.neuron.2015.01.011 25695271

[B21] Donato F. Rompani S. B. Caroni P. (2013). Parvalbumin-expressing basket-cell network plasticity induced by experience regulates adult learning. *Nature* 504 272–276. 10.1038/nature12866 24336286

[B22] Drexel M. Romanov R. A. Wood J. Weger S. Heilbronn R. Wulff P. (2017). Selective silencing of hippocampal parvalbumin interneurons induces development of recurrent spontaneous limbic seizures in mice. *J. Neurosci.* 37 8166–8179. 10.1523/JNEUROSCI.3456-16.2017 28733354 PMC6596787

[B23] Druga R. Salaj M. Al-Redouan A. (2023). Parvalbumin - positive neurons in the neocortex: A review. *Physiol. Res.* 72:S173. 10.33549/physiolres.935005 37565421 PMC10660579

[B24] Dudok B. Klein P. M. Hwaun E. Lee B. R. Yao Z. Fong O. (2021). Alternating sources of perisomatic inhibition during behavior. *Neuron* 109 997–1012.e9. 10.1016/j.neuron.2021.01.003. 33529646 PMC7979482

[B25] Eggermann E. Bucurenciu I. Goswami S. P. Jonas P. (2011). Nanodomain coupling between Ca2+ channels and sensors of exocytosis at fast mammalian synapses. *Nat. Rev. Neurosci.* 13 7–21. 10.1038/nrn3125 22183436 PMC3617475

[B26] Favero M. Sotuyo N. P. Lopez E. Kearney J. A. Goldberg E. M. (2018). A transient developmental window of fast-spiking interneuron dysfunction in a mouse model of dravet syndrome. *J. Neurosci.* 38 7912–7927. 10.1523/JNEUROSCI.0193-18.2018 30104343 PMC6125809

[B27] Fernandez-Ruiz A. Sirota A. Lopes-dos-Santos V. Dupret D. (2023). Over and above frequency: Gamma oscillations as units of neural circuit operations. *Neuron* 111 936–953. 10.1016/j.neuron.2023.02.026 37023717 PMC7614431

[B28] Ferraguti F. Cobden P. Pollard M. Cope D. Shigemoto R. Watanabe M. (2004). Immunolocalization of metabotropic glutamate receptor 1α (mGluR1α) in distinct classes of interneuron in the CA1 region of the rat hippocampus. *Hippocampus* 14 193–215. 10.1002/hipo.10163 15098725

[B29] Freund T. F. Buzsáki G. (1996). Interneurons of the hippocampus. *Hippocampus* 6 347–470. 10.1002/(SICI)1098-106319966:4<347::AID-HIPO1>3.0.CO;2-I 8915675

[B30] Freund T. F. Katona I. (2007). Perisomatic Inhibition. *Neuron* 56 33–42. 10.1016/j.neuron.2007.09.012 17920013

[B31] Fujima S. Sato M. Nakai N. Takumi T. (2025). Parvalbumin interneurons in the insular cortex control social familiarity and emotion recognition. *Cell Rep.* 44:116085. 10.1016/j.celrep.2025.116085 40865515

[B32] Ganter P. Szücs P. Paulsen O. Somogyi P. (2004). Properties of horizontal axo-axonic cells in stratum oriens of the hippocampal CA1 area of rats in vitro. *Hippocampus* 14 232–243. 10.1002/hipo.10170 15098728

[B33] Geiger J. R. P. Lübke J. Roth A. Frotscher M. Jonas P. (1997). Submillisecond AMPA receptor-mediated signaling at a principal neuron–interneuron synapse. *Neuron* 18 1009–1023. 10.1016/S0896-6273(00)80339-6 9208867

[B34] Geiger J. R. P. Melcher T. Koh D.-S. Sakmann B. Seeburg P. H. Jonas P. (1995). Relative abundance of subunit mRNAs determines gating and Ca2+ permeability of AMPA receptors in principal neurons and interneurons in rat CNS. *Neuron* 15 193–204. 10.1016/0896-6273(95)90076-4 7619522

[B35] Gloveli T. Dugladze T. Saha S. Monyer H. Heinemann U. Traub R. D. (2005). Differential involvement of oriens/pyramidale interneurones in hippocampal network oscillations in vitro. *J. Physiol.* 562 131–147. 10.1113/jphysiol.2004.073007 15486016 PMC1665476

[B36] Goel A. Cantu D. A. Guilfoyle J. Chaudhari G. R. Newadkar A. Todisco B. (2018). Impaired perceptual learning in a mouse model of Fragile X syndrome is mediated by parvalbumin neuron dysfunction and is reversible. *Nat. Neurosci.* 21 1404–1411. 10.1038/s41593-018-0231-0 30250263 PMC6161491

[B37] Goldin M. Epsztein J. Jorquera I. Represa A. Ben-Ari Y. Crépel V. (2007). Synaptic kainate receptors tune oriens-lacunosum moleculare interneurons to operate at theta frequency. *J. Neurosci.* 27 9560–9572. 10.1523/JNEUROSCI.1237-07.2007 17804617 PMC6672977

[B38] Gulyás A. I. Megıas M. Emri Z. Freund T. F. (1999). Total number and ratio of excitatory and inhibitory synapses converging onto single interneurons of different types in the CA1 area of the rat hippocampus. *J. Neurosci.* 19 10082–10097. 10.1523/JNEUROSCI.19-22-10082.1999 10559416 PMC6782984

[B39] Hainmueller T. Cazala A. Huang L.-W. Bartos M. (2024). Subfield-specific interneuron circuits govern the hippocampal response to novelty in male mice. *Nat. Commun.* 15:714. 10.1038/s41467-024-44882-3 38267409 PMC10808551

[B40] Halasy K. Buhl E. H. Lörinczi Z. Tamás G. Somogyi P. (1996). Synaptic target selectivity and input of GABAergic basket and bistratified interneurons in the CA1 area of the rat hippocampus. *Hippocampus* 6 306–329. 10.1002/(SICI)1098-106319966:3<306::AID-HIPO8>3.0.CO;2-K 8841829

[B41] He Y. Li J. Zheng W. Liu J. Dong Z. Yang L. (2025). Hypomyelination in autism-associated neuroligin-3 mutant mice impairs parvalbumin interneuron excitability, gamma oscillations, and sensory discrimination. *Nat. Commun.* 16:6382. 10.1038/s41467-025-61455-0 40640134 PMC12246190

[B42] Hefft S. Jonas P. (2005). Asynchronous GABA release generates long-lasting inhibition at a hippocampal interneuron–principal neuron synapse. *Nat. Neurosci.* 8 1319–1328. 10.1038/nn1542 16158066

[B43] Hijazi S. Smit A. B. van Kesteren R. E. (2023). Fast-spiking parvalbumin-positive interneurons in brain physiology and Alzheimer’s disease. *Mol. Psychiatry* 28 4954–4967. 10.1038/s41380-023-02168-y 37419975 PMC11041664

[B44] Ho Y.-Y. Yang Q. Boddu P. Bulkin D. A. Warden M. R. (2025). Infralimbic parvalbumin neural activity facilitates cued threat avoidance. *eLife* 12:R91221. 10.7554/eLife.91221 40168058 PMC11961119

[B45] Hong I. Kim J. Hainmueller T. Kim D. W. Keijser J. Johnson R. C. (2024). Calcium-permeable AMPA receptors govern PV neuron feature selectivity. *Nature* 635 398–405. 10.1038/s41586-024-08027-2 39358515 PMC11560848

[B46] Houser C. R. (2007). “Interneurons of the dentate gyrus: An overview of cell types, terminal fields and neurochemical identity,” in *Progress in brain research*, ed. Scharfman H. E. (Amsterdam: Elsevier), 217–811. 10.1016/S0079-6123(07)63013-1 17765721

[B47] Hu H. Gan J. Jonas P. (2014). Fast-spiking, parvalbumin+ GABAergic interneurons: From cellular design to microcircuit function. *Science* 345:1255263. 10.1126/science.1255263 25082707

[B48] Hu Y. Feng Y. Luo H. Zhu X.-N. Chen S. Yang K. (2025). Dissociation-related behaviors in mice emerge from the inhibition of retrosplenial cortex parvalbumin interneurons. *Cell Rep.* 44:115086. 10.1016/j.celrep.2024.115086 39708317

[B49] Huang Y.-C. Chen H.-C. Lin Y.-T. Lin S.-T. Zheng Q. Abdelfattah A. S. (2024). Dynamic assemblies of parvalbumin interneurons in brain oscillations. *Neuron* 112 2600–2613.e5. 10.1016/j.neuron.2024.05.015. 38955183 PMC12553520

[B50] Inan M. Zhao M. Manuszak M. Karakaya C. Rajadhyaksha A. M. Pickel V. M. (2016). Energy deficit in parvalbumin neurons leads to circuit dysfunction, impaired sensory gating and social disability. *Neurobiol. Dis.* 93 35–46. 10.1016/j.nbd.2016.04.004 27105708

[B51] Jang H. J. Chung H. Rowland J. M. Richards B. A. Kohl M. M. Kwag J. (2020). Distinct roles of parvalbumin and somatostatin interneurons in gating the synchronization of spike times in the neocortex. *Sci. Adv.* 6:eaay5333. 10.1126/sciadv.aay5333 32426459 PMC7176419

[B52] Jinno S. Klausberger T. Marton L. F. Dalezios Y. Roberts J. D. B. Fuentealba P. (2007). Neuronal diversity in GABAergic long-range projections from the hippocampus. *J. Neurosci.* 27 8790–8804. 10.1523/JNEUROSCI.1847-07.2007 17699661 PMC2270609

[B53] Karube F. Kubota Y. Kawaguchi Y. (2004). Axon branching and synaptic bouton phenotypes in GABAergic nonpyramidal cell subtypes. *J. Neurosci.* 24 2853–2865. 10.1523/JNEUROSCI.4814-03.2004 15044524 PMC6729850

[B54] Kaufhold D. Casas E. M. de las Ocaña-Fernández M. D. Á Cazala A. Yuan M. Kulik A. (2024). Spine plasticity of dentate gyrus parvalbumin-positive interneurons is regulated by experience. *Cell Rep.* 43:113806. 10.1016/j.celrep.2024.113806 38377001

[B55] Kim H. Ährlund-Richter S. Wang X. Deisseroth K. Carlén M. (2016). Prefrontal parvalbumin neurons in control of attention. *Cell* 164 208–218. 10.1016/j.cell.2015.11.038 26771492 PMC4715187

[B56] Kim H. K. Gschwind T. Nguyen T. M. Bui A. D. Felong S. Ampig K. (2020). Optogenetic intervention of seizures improves spatial memory in a mouse model of chronic temporal lobe epilepsy. *Epilepsia* 61 561–571. 10.1111/epi.16445 32072628 PMC7708390

[B57] Klausberger T. Magill P. J. Márton L. F. Roberts J. D. B. Cobden P. M. Buzsáki G. (2003). Brain-state- and cell-type-specific firing of hippocampal interneurons in vivo. *Nature* 421 844–848. 10.1038/nature01374 12594513

[B58] Klausberger T. Márton L. F. Baude A. Roberts J. D. B. Magill P. J. Somogyi P. (2004). Spike timing of dendrite-targeting bistratified cells during hippocampal network oscillations in vivo. *Nat. Neurosci.* 7 41–47. 10.1038/nn1159 14634650

[B59] Klausberger T. Somogyi P. (2008). Neuronal diversity and temporal dynamics: The unity of hippocampal circuit operations. *Science* 321 53–57. 10.1126/science.1149381 18599766 PMC4487503

[B60] Koh D. S. Geiger J. R. Jonas P. Sakmann B. (1995). Ca(2+)-permeable AMPA and NMDA receptor channels in basket cells of rat hippocampal dentate gyrus. *J. Physiol.* 485 383–402. 10.1113/jphysiol.1995.sp020737 7545230 PMC1158000

[B61] Korotkova T. Fuchs E. C. Ponomarenko A. von Engelhardt J. Monyer H. (2010). NMDA receptor ablation on parvalbumin-positive interneurons impairs hippocampal synchrony, spatial representations, and working memory. *Neuron* 68 557–569. 10.1016/j.neuron.2010.09.017 21040854

[B62] Krook-Magnuson E. Armstrong C. Oijala M. Soltesz I. (2013). On-demand optogenetic control of spontaneous seizures in temporal lobe epilepsy. *Nat. Commun.* 4:1376. 10.1038/ncomms2376 23340416 PMC3562457

[B63] Kubota Y. (2014). Untangling GABAergic wiring in the cortical microcircuit. *Curr. Opin. Neurobiol.* 26 7–14. 10.1016/j.conb.2013.10.003 24650498

[B64] Lee S.-H. Marchionni I. Bezaire M. Varga C. Danielson N. Lovett-Barron M. (2014). Parvalbumin-Positive basket cells differentiate among hippocampal pyramidal cells. *Neuron* 82 1129–1144. 10.1016/j.neuron.2014.03.034 24836505 PMC4076442

[B65] Lévesque M. Chen L.-Y. Etter G. Shiri Z. Wang S. Williams S. (2019). Paradoxical effects of optogenetic stimulation in mesial temporal lobe epilepsy. *Ann. Neurol.* 86 714–728. 10.1002/ana.25572 31393618

[B66] Li X. G. Somogyi P. Tepper J. M. Buzsáki G. (1992). Axonal and dendritic arborization of an intracellularly labeled chandelier cell in the CA1 region of rat hippocampus. *Exp. Brain Res.* 90 519–525. 10.1007/BF00230934 1385200

[B67] Lorincz A. Nusser Z. (2008). Cell-Type-Dependent molecular composition of the axon initial segment. *J. Neurosci.* 28 14329–14340. 10.1523/JNEUROSCI.4833-08.2008 19118165 PMC2628579

[B68] Maas D. A. Eijsink V. D. Spoelder M. van Hulten J. A. De Weerd P. Homberg J. R. (2020). Interneuron hypomyelination is associated with cognitive inflexibility in a rat model of schizophrenia. *Nat. Commun.* 11:2329. 10.1038/s41467-020-16218-4 32393757 PMC7214427

[B69] Marissal T. (2021). An inventory of basic research in temporal lobe epilepsy. *Rev. Neurol.* 177 1069–1081. 10.1016/j.neurol.2021.02.390 34176659

[B70] Marissal T. Salazar R. F. Bertollini C. Mutel S. De Roo M. Rodriguez I. (2018). Restoring wild-type-like CA1 network dynamics and behavior during adulthood in a mouse model of schizophrenia. *Nat. Neurosci.* 21 1412–1420. 10.1038/s41593-018-0225-y 30224804 PMC6978142

[B71] Matringhen C. Vigier A. Bourtouli N. Michel F. J. Marissal T. (2025). Minimally-invasive manipulation of spared and hypoactive interneurons reduces CA1 synchronization and nonspatial behavior alterations in epilepsy models. *Neurobiol. Dis.* 217:107155. 10.1016/j.nbd.2025.107155 41151694

[B72] McBain C. J. DiChiara T. J. Kauer J. A. (1994). Activation of metabotropic glutamate receptors differentially affects two classes of hippocampal interneurons and potentiates excitatory synaptic transmission. *J. Neurosci.* 14 4433–4445. 10.1523/JNEUROSCI.14-07-04433.1994 7517996 PMC6577047

[B73] Meechan D. W. Tucker E. S. Maynard T. M. LaMantia A.-S. (2012). Cxcr4 regulation of interneuron migration is disrupted in 22q11.2 deletion syndrome. *PNAS* 109 18601–18606. 10.1073/pnas.1211507109 23091025 PMC3494945

[B74] Micheva K. D. Kiraly M. Perez M. M. Madison D. V. (2021). Extensive structural remodeling of the axonal arbors of parvalbumin basket cells during development in mouse neocortex. *J. Neurosci.* 41 9326–9339. 10.1523/JNEUROSCI.0871-21.2021 34583957 PMC8580153

[B75] Mukherjee A. Carvalho F. Eliez S. Caroni P. (2019). Long-Lasting rescue of network and cognitive dysfunction in a genetic schizophrenia model. *Cell* 178 1387–1402.e14. 10.1016/j.cell.2019.07.023. 31474363

[B76] Nguyen R. Morrissey M. D. Mahadevan V. Cajanding J. D. Woodin M. A. Yeomans J. S. (2014). Parvalbumin and GAD65 interneuron inhibition in the ventral hippocampus induces distinct behavioral deficits relevant to schizophrenia. *J. Neurosci.* 34 14948–14960. 10.1523/JNEUROSCI.2204-14.2014 25378161 PMC4220027

[B77] Ogiwara I. Miyamoto H. Morita N. Atapour N. Mazaki E. Inoue I. (2007). Nav1.1 localizes to axons of parvalbumin-positive inhibitory interneurons: A circuit basis for epileptic seizures in mice carrying an scn1a gene mutation. *J. Neurosci.* 27 5903–5914. 10.1523/JNEUROSCI.5270-06.2007 17537961 PMC6672241

[B78] Ognjanovski N. Schaeffer S. Wu J. Mofakham S. Maruyama D. Zochowski M. (2017). Parvalbumin-expressing interneurons coordinate hippocampal network dynamics required for memory consolidation. *Nat. Commun.* 8:15039. 10.1038/ncomms15039 28382952 PMC5384212

[B79] Onorato I. Tzanou A. Schneider M. Uran C. Broggini A. C. Vinck M. (2025). Distinct roles of PV and Sst interneurons in visually induced gamma oscillations. *Cell Rep.* 44:115385. 10.1016/j.celrep.2025.115385 40048428

[B80] Packer A. M. Yuste R. (2011). Dense, unspecific connectivity of neocortical parvalbumin-positive interneurons: A canonical microcircuit for inhibition? *J. Neurosci.* 31 13260–13271. 10.1523/JNEUROSCI.3131-11.2011 21917809 PMC3178964

[B81] Pang Z. P. Melicoff E. Padgett D. Liu Y. Teich A. F. Dickey B. F. (2006). Synaptotagmin-2 is essential for survival and contributes to Ca2+ triggering of neurotransmitter release in central and neuromuscular synapses. *J. Neurosci.* 26 13493–13504. 10.1523/JNEUROSCI.3519-06.2006 17192432 PMC6674714

[B82] Parker M. M. Rubin J. E. Huang C. (2025). State modulation in spatial networks with three interneuron subtypes. *Sci. Adv.* 11:eads9134. 10.1126/sciadv.ads9134 40561011 PMC13108820

[B83] Patz S. Grabert J. Gorba T. Wirth M. J. Wahle P. (2004). Parvalbumin expression in visual cortical interneurons depends on neuronal activity and TrkB ligands during an early period of postnatal development. *Cereb. Cortex* 14 342–351. 10.1093/cercor/bhg132 14754872

[B84] Paul A. Crow M. Raudales R. He M. Gillis J. Huang Z. J. (2017). Transcriptional architecture of synaptic communication delineates GABAergic neuron identity. *Cell* 171 522–539.e20. 10.1016/j.cell.2017.08.032. 28942923 PMC5772785

[B85] Pawelzik H. Hughes D. I. Thomson A. M. (2002). Physiological and morphological diversity of immunocytochemically defined parvalbumin- and cholecystokinin-positive interneurones in CA1 of the adult rat hippocampus. *J. Comp. Neurol.* 443 346–367. 10.1002/cne.10118 11807843

[B86] Pedroncini O. Federman N. Marin-Burgin A. (2024). Lateral entorhinal cortex afferents reconfigure the activity in piriform cortex circuits. *Proc. Natl. Acad. Sci. U. S. A.* 121:e2414038121. 10.1073/pnas.2414038121 39570314 PMC11621770

[B87] Pelkey K. A. Chittajallu R. Craig M. T. Tricoire L. Wester J. C. McBain C. J. (2017). Hippocampal GABAergic inhibitory interneurons. *Physiol. Rev.* 97 1619–1747. 10.1152/physrev.00007.2017 28954853 PMC6151493

[B88] Peng Y. Barreda Tomas F. J. Pfeiffer P. Drangmeister M. Schreiber S. Vida I. (2021). Spatially structured inhibition defined by polarized parvalbumin interneuron axons promotes head direction tuning. *Sci. Adv.* 7:eabg4693. 10.1126/sciadv.abg4693 34134979 PMC8208710

[B89] Pignataro A. Krashia P. De Introna M. Nobili A. Sabetta A. Stabile F. (2023). Chemogenetic rectification of the inhibitory tone onto hippocampal neurons reverts autistic-like traits and normalizes local expression of estrogen receptors in the Ambra1+/- mouse model of female autism. *Transl. Psychiatry* 13:63. 10.1038/s41398-023-02357-x 36804922 PMC9941573

[B90] Proddutur A. Nguyen S. Yeh C.-W. Gupta A. Santhakumar V. (2023). Reclusive chandeliers: Functional isolation of dentate axo-axonic cells after experimental status epilepticus. *Prog. Neurobiol.* 231:102542. 10.1016/j.pneurobio.2023.102542 37898313 PMC10842856

[B91] Qi C. Sima W. Mao H. Hu E. Ge J. Deng M. (2025). Anterior cingulate cortex parvalbumin and somatostatin interneurons shape social behavior in male mice. *Nat. Commun.* 16:4156. 10.1038/s41467-025-59473-z 40320404 PMC12050299

[B92] Raudales R. Kim G. Kelly S. M. Hatfield J. Guan W. Zhao S. (2024). Specific and comprehensive genetic targeting reveals brain-wide distribution and synaptic input patterns of GABAergic axo-axonic interneurons. *eLife* 13:R93481. 10.7554/eLife.93481 39012795 PMC11251723

[B93] Raven F. Aton S. J. (2021). The engram’s dark horse: How interneurons regulate state-dependent memory processing and plasticity. *Front. Neural Circuits* 15:750541. 10.3389/fncir.2021.750541 34588960 PMC8473837

[B94] Rossignol E. Kruglikov I. van den Maagdenberg A. M. J. M. Rudy B. Fishell G. (2013). CaV2.1 ablation in cortical interneurons selectively impairs fast-spiking basket cells and causes generalized seizures. *Ann. Neurol.* 74 209–222. 10.1002/ana.23913 23595603 PMC3849346

[B95] Roth B. L. (2016). DREADDs for neuroscientists. *Neuron* 89 683–694. 10.1016/j.neuron.2016.01.040 26889809 PMC4759656

[B96] Royer S. Zemelman B. V. Losonczy A. Kim J. Chance F. Magee J. C. (2012). Control of timing, rate and bursts of hippocampal place cells by dendritic and somatic inhibition. *Nat. Neurosci.* 15 769–775. 10.1038/nn.3077 22446878 PMC4919905

[B97] Ruden J. B. Dugan L. L. Konradi C. (2021). Parvalbumin interneuron vulnerability and brain disorders. *Neuropsychopharmacology* 46 279–287. 10.1038/s41386-020-0778-9 32722660 PMC7852528

[B98] Rudy B. Fishell G. Lee S. Hjerling-Leffler J. (2011). Three groups of interneurons account for nearly 100% of neocortical GABAergic neurons. *Dev. Neurobiol.* 71:45. 10.1002/dneu.20853 21154909 PMC3556905

[B99] Rupert D. D. Shea S. D. (2022). Parvalbumin-Positive interneurons regulate cortical sensory plasticity in adulthood and development through shared mechanisms. *Front. Neural Circuits* 16:886629. 10.3389/fncir.2022.886629 35601529 PMC9120417

[B100] Sauer J.-F. Strüber M. Bartos M. (2015). Impaired fast-spiking interneuron function in a genetic mouse model of depression. *eLife* 4:e04979. 10.7554/eLife.04979 25735038 PMC4374525

[B101] Shuman T. Aharoni D. Cai D. J. Lee C. R. Chavlis S. Page-Harley L. (2020). Breakdown of spatial coding and interneuron synchronization in epileptic mice. *Nat. Neurosci.* 23 229–238. 10.1038/s41593-019-0559-0 31907437 PMC7259114

[B102] Sohal V. S. Zhang F. Yizhar O. Deisseroth K. (2009). Parvalbumin neurons and gamma rhythms enhance cortical circuit performance. *Nature* 459 698–702. 10.1038/nature07991 19396159 PMC3969859

[B103] Sommeijer J.-P. Levelt C. N. (2012). Synaptotagmin-2 is a reliable marker for parvalbumin positive inhibitory boutons in the mouse visual cortex. *PLoS One* 7:e35323. 10.1371/journal.pone.0035323 22539967 PMC3335159

[B104] Somogyi P. (1977). A specific ‘axo-axonal’ interneuron in the visual cortex of the rat. *Brain Res.* 136 345–350. 10.1016/0006-8993(77)90808-3 922488

[B105] Stedehouder J. Couey J. J. Brizee D. Hosseini B. Slotman J. A. Dirven C. M. F. (2017). Fast-spiking parvalbumin interneurons are frequently myelinated in the cerebral cortex of mice and humans. *Cereb. Cortex* 27 5001–5013. 10.1093/cercor/bhx203 28922832

[B106] Stokes C. C. A. Isaacson J. S. (2010). From dendrite to soma: Dynamic routing of inhibition by complementary interneuron microcircuits in olfactory cortex. *Neuron* 67 452–465. 10.1016/j.neuron.2010.06.029 20696382 PMC2922014

[B107] Tai C. Abe Y. Westenbroek R. E. Scheuer T. Catterall W. A. (2014). Impaired excitability of somatostatin- and parvalbumin-expressing cortical interneurons in a mouse model of Dravet syndrome. *Proc. Natl. Acad. Sci. U. S. A.* 111 E3139–E3148. 10.1073/pnas.1411131111 25024183 PMC4121787

[B108] Taniguchi H. He M. Wu P. Kim S. Paik R. Sugino K. (2011). A resource of cre driver lines for genetic targeting of GABAergic neurons in cerebral cortex. *Neuron* 71 995–1013. 10.1016/j.neuron.2011.07.026 21943598 PMC3779648

[B109] Tiwari P. Davoudian P. A. Kapri D. Vuruputuri R. M. Karaba L. A. Sharma M. (2024). Ventral hippocampal parvalbumin interneurons gate the acute anxiolytic action of the serotonergic psychedelic DOI. *Neuron* 112 3697–3714.e6. 10.1016/j.neuron.2024.08.016. 39321791 PMC11581910

[B110] Tomasella E. Bechelli L. Ogando M. B. Mininni C. Di Guilmi M. N. De Fino F. (2018). Deletion of dopamine D2 receptors from parvalbumin interneurons in mouse causes schizophrenia-like phenotypes. *Proc. Natl. Acad. Sci. U. S. A.* 115 3476–3481. 10.1073/pnas.1719897115 29531031 PMC5879696

[B111] Tremblay R. Lee S. Rudy B. (2016). GABAergic interneurons in the neocortex: From cellular properties to circuits. *Neuron* 91:260. 10.1016/j.neuron.2016.06.033 27477017 PMC4980915

[B112] Tricoire L. Pelkey K. A. Erkkila B. E. Jeffries B. W. Yuan X. McBain C. J. (2011). A blueprint for the spatiotemporal origins of mouse hippocampal interneuron diversity. *J. Neurosci.* 31 10948–10970. 10.1523/JNEUROSCI.0323-11.2011 21795545 PMC3163481

[B113] Tsien J. Z. Chen D. F. Gerber D. Tom C. Mercer E. H. Anderson D. J. (1996). Subregion- and cell type–restricted gene knockout in mouse brain. *Cell* 87 1317–1326. 10.1016/S0092-8674(00)81826-7 8980237

[B114] Tukker J. J. Lasztóczi B. Katona L. Roberts J. D. B. Pissadaki E. K. Dalezios Y. (2013). Distinct dendritic arborization and in vivo firing patterns of parvalbumin-expressing basket cells in the hippocampal area CA3. *J. Neurosci.* 33 6809–6825. 10.1523/JNEUROSCI.5052-12.2013 23595740 PMC4473055

[B115] Tzilivaki A. Tukker J. J. Maier N. Poirazi P. Sammons R. P. Schmitz D. (2023). Hippocampal GABAergic interneurons and memory. *Neuron* 111 3154–3175. 10.1016/j.neuron.2023.06.016 37467748 PMC10593603

[B116] Udakis M. Pedrosa V. Chamberlain S. E. L. Clopath C. Mellor J. R. (2020). Interneuron-specific plasticity at parvalbumin and somatostatin inhibitory synapses onto CA1 pyramidal neurons shapes hippocampal output. *Nat. Commun.* 11:4395. 10.1038/s41467-020-18074-8 32879322 PMC7467931

[B117] Uhlhaas P. J. Singer W. (2010). Abnormal neural oscillations and synchrony in schizophrenia. *Nat. Rev. Neurosci.* 11 100–113. 10.1038/nrn2774 20087360

[B118] Upadhya D. Hattiangady B. Castro O. W. Shuai B. Kodali M. Attaluri S. (2019). Human induced pluripotent stem cell-derived MGE cell grafting after status epilepticus attenuates chronic epilepsy and comorbidities via synaptic integration. *PNAS* 116 287–296. 10.1073/pnas.1814185115 30559206 PMC6320542

[B119] Varga C. Golshani P. Soltesz I. (2012). Frequency-invariant temporal ordering of interneuronal discharges during hippocampal oscillations in awake mice. *Proc. Natl. Acad. Sci. U. S. A.* 109 E2726–E2734. 10.1073/pnas.1210929109 23010933 PMC3479571

[B120] Varga C. Oijala M. Lish J. Szabo G. G. Bezaire M. Marchionni I. (2014). Functional fission of parvalbumin interneuron classes during fast network events. *eLife* 3:e04006. 10.7554/eLife.04006 25375253 PMC4270094

[B121] Viney T. J. Lasztoczi B. Katona L. Crump M. G. Tukker J. J. Klausberger T. (2013). Network state-dependent inhibition of identified hippocampal CA3 axo-axonic cells in vivo. *Nat. Neurosci.* 16 1802–1811. 10.1038/nn.3550 24141313 PMC4471148

[B122] Volitaki E. Forro T. Li K. Nevian T. Ciocchi S. (2024). Activity of ventral hippocampal parvalbumin interneurons during anxiety. *Cell Rep.* 43:114295. 10.1016/j.celrep.2024.114295 38796850

[B123] Wang B. Ke W. Guang J. Chen G. Yin L. Deng S. (2016). Firing frequency maxima of fast-spiking neurons in human, monkey, and mouse neocortex. *Front. Cell. Neurosci.* 10:239. 10.3389/fncel.2016.00239 27803650 PMC5067378

[B124] Wang X.-J. Tegnér J. Constantinidis C. Goldman-Rakic P. S. (2004). Division of labor among distinct subtypes of inhibitory neurons in a cortical microcircuit of working memory. *Proc. Natl. Acad. Sci. U. S. A.* 101:1368. 10.1073/pnas.0305337101 14742867 PMC337059

[B125] Wang Y. Zhang P. Wyskiel D. R. (2016). Chandelier cells in functional and dysfunctional neural circuits. *Front. Neural Circuits* 10:33. 10.3389/fncir.2016.00033 27199673 PMC4854894

[B126] Wick Z. C. Leintz C. H. Xamonthiene C. Huang B. H. Krook-Magnuson E. (2017). Axonal sprouting in commissurally projecting parvalbumin-expressing interneurons. *J. Neurosci. Res.* 95:2336. 10.1002/jnr.24011 28151564 PMC5540851

[B127] Wöhr M. Orduz D. Gregory P. Moreno H. Khan U. Vörckel K. J. (2015). Lack of parvalbumin in mice leads to behavioral deficits relevant to all human autism core symptoms and related neural morphofunctional abnormalities. *Trans. Psychiatry* 5:e525. 10.1038/tp.2015.19 25756808 PMC4354349

[B128] Xia F. Richards B. A. Tran M. M. Josselyn S. A. Takehara-Nishiuchi K. Frankland P. W. (2017). Parvalbumin-positive interneurons mediate neocortical-hippocampal interactions that are necessary for memory consolidation. *eLife* 6:e27868. 10.7554/eLife.27868 28960176 PMC5655147

[B129] Yamada J. Ohgomori T. Jinno S. (2015). Perineuronal nets affect parvalbumin expression in GABAergic neurons of the mouse hippocampus. *Eur. J. Neurosci.* 41 368–378. 10.1111/ejn.12792 25411016

[B130] Yang J.-W. Prouvot P.-H. Reyes-Puerta V. Stüttgen M. C. Stroh A. Luhmann H. J. (2017). Optogenetic modulation of a minor fraction of parvalbumin-positive interneurons specifically affects spatiotemporal dynamics of spontaneous and sensory-evoked activity in mouse somatosensory cortex in vivo. *Cereb. Cortex* 27:5784. 10.1093/cercor/bhx261 29040472 PMC5939210

[B131] Yen T.-Y. Huang X. MacLaren D. A. A. Schlesiger M. I. Monyer H. Lien C.-C. (2022). Inhibitory projections connecting the dentate gyri in the two hemispheres support spatial and contextual memory. *Cell Rep.* 39:110831. 10.1016/j.celrep.2022.110831 35584671

[B132] Zaitsev A. V. Povysheva N. V. Lewis D. A. Krimer L. S. (2007). P/Q-Type, but not N-Type, calcium channels mediate GABA release from fast-spiking interneurons to pyramidal cells in rat prefrontal cortex. *J. Neurophysiol.* 97 3567–3573. 10.1152/jn.01293.2006 17329622

[B133] Zhang N. Hu B.-W. Li X.-M. Huang H. (2025). Rethinking parvalbumin: From passive marker to active modulator of hippocampal circuits. *IBRO Neurosci. Rep.* 19 760–773. 10.1016/j.ibneur.2025.10.005 41211047 PMC12595124

[B134] Znamenskiy P. Kim M.-H. Muir D. R. Iacaruso M. F. Hofer S. B. Mrsic-Flogel T. D. (2024). Functional specificity of recurrent inhibition in visual cortex. *Neuron* 112 991–1000.e8. 10.1016/j.neuron.2023.12.013 38244539 PMC7618320

